# Characterization of Differentially Detectable Mycobacterium tuberculosis in the Sputum of Subjects with Drug-Sensitive or Drug-Resistant Tuberculosis before and after Two Months of Therapy

**DOI:** 10.1128/AAC.00608-21

**Published:** 2021-07-16

**Authors:** Kayvan Zainabadi, Kathleen Frances Walsh, Stalz Charles Vilbrun, Laurent Daniel Mathurin, Myung Hee Lee, Kohta Saito, Saurabh Mishra, Oksana Ocheretina, Jean William Pape, Carl Nathan, Daniel W. Fitzgerald

**Affiliations:** a Center for Global Health, Weill Cornell Medicine, New York, New York, USA; b Department of Microbiology & Immunology, Weill Cornell Medicine, New York, New York, USA; c Division of General Internal Medicine, Department of Medicine, Weill Cornell Medicine, New York, New York, USA; d Les Centres GHESKIO, Port-au-Prince, Haiti

**Keywords:** *Mycobacterium tuberculosis*, diagnostics, multidrug resistance, pulmonary infection, viable but nonculturable

## Abstract

Standard methods for enumerating Mycobacterium tuberculosis in patient sputum can miss large populations of viable M. tuberculosis cells that are unable to grow either on solid medium or in liquid medium unless the medium has been extensively diluted. Because these bacteria can be detected in liquid medium after limiting dilution, they have been termed differentially culturable or differentially detectable M. tuberculosis (DD-*Mtb*). Treatment with isoniazid (H), rifampin (R), pyrazinamide (Z), and ethambutol (E) (HRZE) for 1 to 2 weeks has been shown to increase the representation of DD-*Mtb* in the sputum of drug-sensitive (DS) tuberculosis (TB) patients. However, little is known about DD-*Mtb* after longer periods of treatment with HRZE or in patients with drug-resistant (DR) TB who receive second-line therapies. Here, we measured the proportion of DD-*Mtb* cells in the sputum of 47 subjects, 29 with DS TB and 18 with DR TB, before initiation of treatment and at 2 weeks and 2 months thereafter. Prior to treatment, DD-*Mtb* cells represented the majority of M. tuberculosis cells in the sputum of 21% of subjects with DS TB, and this proportion rose to 65% after 2 weeks of treatment with first-line drugs. In subjects with DR TB, DD-*Mtb* cells were found in the sputum of 29% of subjects prior to treatment initiation, and this proportion remained steady at 31% after 2 weeks of treatment with second-line drugs. By 2 months, DD-*Mtb* cells were detected in the sputum of only 2/15 (13.3%) subjects with DS TB and in 0/15 of subjects with DR TB. One of the DS subjects whose sputum was positive for DD-*Mtb* at month 2 later experienced treatment failure.

## INTRODUCTION

In 2019, 10 million cases of tuberculosis (TB) led to approximately 1.4 million deaths, making TB the leading cause of death from a single infectious agent prior to COVID-19 (1). TB exerts a devastating toll in the developing world, where more than 95% of deaths occur, and is projected to lead to an economic loss of U.S. $1 trillion from 2015 to 2030 (2). New drug regimens are needed to shorten the currently required 6-month treatment protocol for drug-sensitive (DS) TB and to combat drug-resistant (DR) TB.

To evaluate the efficacy of new drug regimens in phase I/II early bactericidal activity (EBA) trials, viable Mycobacterium tuberculosis cells are quantified from the sputum of pulmonary TB patients during the first 2 to 8 weeks of therapy. The gold-standard method for enumerating M. tuberculosis is culturing sputum on solid agar and counting the number of CFU. This approach is often supplemented or replaced by assessing time to positivity (TTP) of sputum samples in an automated liquid culture system that measures the rate of depletion of oxygen from culture medium as a proxy for M. tuberculosis growth and quantity. These techniques may miss subpopulations of M. tuberculosis that are incapable of growing on solid medium or in liquid medium if the suspension has not been extensively diluted (3–12). These bacteria have been called by the interchangeable terms “viable but nonculturable” (VBNC-*Mtb*) (13), “differentially culturable” (DCTB) (10), and “differentially detectable” M. tuberculosis (DD-*Mtb*) (8). Here, we refer to these bacteria as “DD-*Mtb*” because they are often culturable and because they can sometimes be identified by methods other than culture, such as by inoculation into an experimental host. The role of limiting dilution in detection of DD-*Mtb* has been linked to imposition of a delay in onset of replication, which allows for repair of oxidative injury (K. Saito, S. Mishra, T. Warrier, N. Cicchetti, J. Mi, E. Weber, J. Roberts, A. Gouzy, E. Kaplan, C. D. Brown, B. Gold, and C. Nathan, unpublished data). Notably, DD-*Mtb* cells show profound phenotypic tolerance to anti-TB chemotherapy (8, 14, 15).

DD-*Mtb* bacteria were suspected to exist in humans when lung homogenates prepared at autopsy detected no M. tuberculosis based on CFU, but aliquots of the same homogenates caused TB in guinea pigs (16). These suspicions were validated in 2010 when Mukamolova et al. (9) reported that DD-*Mtb* constituted 80 to 99.9% of the viable M. tuberculosis bacteria in sputa from 20 of 25 (80%) treatment-naive TB patients, as revealed by comparing CFU counts to the most probable number (MPN) estimation from liquid medium using a limiting dilution (LD) assay. Treatment with rifampin for 7 to 11 days reduced the number of M. tuberculosis bacteria detected by CFU in the sputa to a much greater extent than it reduced M. tuberculosis bacteria detected by the MPN-LD method, indicating proportionate enrichment of DD-*Mtb* populations during therapy (9). Similarly, Chengalroyen et al. (10) reported that DD-*Mtb* represented the majority of viable M. tuberculosis cells in the sputa of >70% of 110 treatment-naive TB patients in South Africa. In both studies, DD-*Mtb* populations were detectable in some specimens only when the LD assay was performed in medium supplemented with culture filtrate (CF), that is, cell-free medium from a growing culture of a laboratory strain of M. tuberculosis. However, in the studies of Chengalroyen et al. (10) and McAulay et al. (11), MPN-LD assays performed with CF impaired the detection of DD-*Mtb* from some sputum samples (17).

With methodological and statistical methods designed to increase stringency (8), sputum samples from 21% of treatment-naive Haitian subjects with TB were found to be dominated by DD-*Mtb*, and this proportion increased to 69% after 2 weeks of isoniazid (H), rifampin (R), pyrazinamide (Z), and ethambutol (E) (HRZE) treatment (11). That study left unanswered the questions of how long DD-*Mtb* populations persist during treatment and how treatments other than HRZE may affect their persistence and/or generation.

Collectively, these findings suggest that not taking DD-*Mtb* into account could complicate interpretation of EBA trials of investigational TB drugs and contribute to the ambiguous clinical significance of sputum conversion—the apparent elimination of viable M. tuberculosis bacteria— after 2 months of treatment (5, 8, 9, 11, 14, 15). The present study is meant to help lay the groundwork for filling the following knowledge gaps about DD-*Mtb*. Does their prevalence differ between specimens from subjects with DS or DR TB? Is the impact of HRZE on their prevalence different from the impact of other treatment regimens? What happens to DD-*Mtb* bacteria in sputum when measured at 2 weeks and at 2 months of treatment, at a later time point than those in earlier studies? To address these questions, we used the methods of Saito et al. (8) and McAulay et al. (11) to study additional subjects with DS TB and those with DR TB, to perform DD-*Mtb* tests before treatment and at 2 weeks and 2 months after the onset of treatment, and to monitor the clinical outcome at 6 months.

## RESULTS

### Patient characteristics.

Between May 2018 and September 2020, we enrolled 29 participants with DS TB and 18 participants with DR TB. Their characteristics are described in Table 1. The prevalences of bilateral disease, dyspnea (grade 2+), and prior treatment with first-line drugs were significantly higher at time of enrollment for participants with DR versus those with DS TB (Table 1).

**TABLE 1:** Characteristics of the drug-sensitive and drug-resistant TB cohorts at time of enrollment

Characteristic^*b*^	Cohort^*c*^		*P*
	DS (*n* = 29)	DR (*n* = 18)	
Male (% [*n*])	62.1 (18)	50 (9)	0.546
HIV+ (% [*n*])^*a*^	3.4 (1)	22.2 (4)	0.063
Presence of cavities (% [*n*])	48.3 (14)	44.4 (8)	1.0
Bilateral disease (% [*n*])	31.0 (9)	72.2 (13)	0.008
Prior first-line TB treatment (% [*n*])	0 (0)	83.3 (15)	<0.001
Blood-tinged sputum (% [*n*])	0 (0)	11.1 (2)	0.142
Pleuritic chest pain (% [*n*])	34.5 (10)	55.6 (10)	0.226
Fever (% [*n*])	34.5 (10)	11.1 (2)	0.095
Age in yrs (median [IQR])	33 (28–37)	32 (28–41)	0.793
Hemoglobin, g/dl (median [IQR])	10.7 (9.8–11.3)	11.0 (10.3–11.5)	0.456
Creatinine, mg/dl (median [IQR])	0.7 (0.6–0.8)	0.6 (0.5–0.7)	0.159
Pulse oximetry, % (median [IQR])	97 (95–98)	98 (97–99)	0.113
BMI (median [IQR])	19.0 (17.1–21.0)	17.7 (16.8–19.6)	0.381
Dyspnea (% [*n*])			<0.001
Not present	44.8 (13)	16.7 (3)	
Grade 1	55.2 (16)	22.2 (4)	
Grade 2	0 (0)	55.6 (10)	
Grade 3	0 (0)	5.6 (1)	
Cough (% [*n*])			0.074
Not present	0 (0)	5.6 (1)	
Grade 1	89.7 (26)	66.7 (12)	
Grade 2	10.3 (3)	22.2 (4)	
Grade 3	0 (0)	5.6 (1)	
Xpert positivity (% [*n*])			0.948
Very low	3.4 (1)	0 (0)	
Low	13.8 (4)	11.1 (2)	
Medium	37.9 (11)	50 (9)	
High	44.8 (13)	38.9 (7)	

a All HIV+ participants were on antiretroviral therapy at time of enrollment and during the duration of this study.

b IQR, interquartile range; BMI, body mass index.

c DS, drug susceptible; DR, drug resistant.

Of the 18 DR participants, 15 had prior treatment with first-line anti-TB drugs. All isolates were resistant to rifampin (with isolates from 14 subjects showing resistance to rifabutin as well); 17 also showed resistance to isoniazid, and 15 were resistant to additional drugs besides rifamycins and isoniazid (see Table S1 in the supplemental material).

### Proportion of TB subjects with DD-*Mtb* before and after initiation of therapy.

M. tuberculosis numbers were quantified from the sputa of DS and DR TB subjects in three different ways, as follows: (i) CFU counting using solid agar plates, (ii) MPN using a limiting dilution (MPN-LD) assay without culture filtrate (MPN^−CF^), and (iii) MPN-LD assay with 50% sterile culture filtrate (MPN^+CF^). DD-*Mtb* was said to be present when the CFU value was less than the lower bound of the 95% confidence interval of the MPN^−CF^ or MPN^+CF^ value. Some sample collections were missed because of civic unrest and the COVID-19 pandemic, and some samples were lost to contamination, so that day 0 and week 2 data were both available for 23/29 and 17/18 subjects with DS and DR TB, respectively; and month 2 data were available for 15/29 and 15/18 subjects with DS and DR TB, respectively.

**FIG 1:**
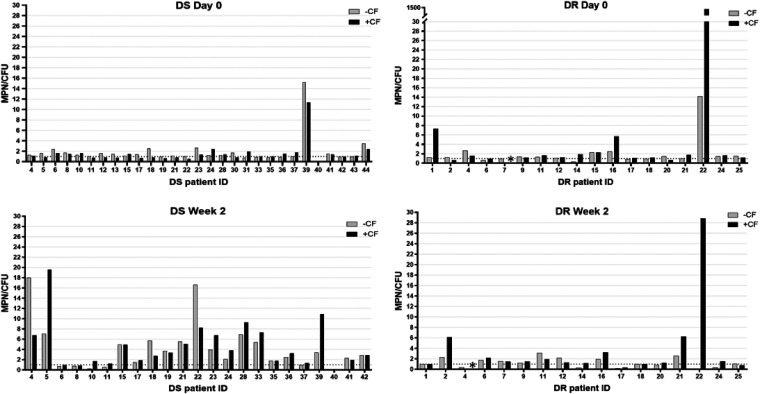
Ratio of most probable number (MPN) to CFU (MPN/CFU), with and without culture filtrate (CF), for sputum from subjects with drug-sensitive (DS) and drug-resistant (DR) TB at day 0 and week 2 post initiation of therapy. The dashed line represents an MPN/CFU ratio of 1. Raw numbers can be found in Table S4 in the supplemental material. An asterisk (*) indicates data not available due to contamination.

The proportion of participants whose sputa were DD-*Mtb* positive was comparable before onset of therapy in treatment-naive subjects with DS TB (20.7%) and in those with DR TB before administration of second-line therapy (29.4%) (Fig. 1 and Table 2). This was apparent when performing the MPN-LD assay without CF (17.2% versus 22.2% positivity for DD-*Mtb* in the sputa of subjects with DS and DR TB, respectively), although when the MPN-LD assay was performed with CF, a 2.3-fold higher proportion of subjects with DR TB showed presence of DD-*Mtb* in their sputa in comparison to subjects with DS TB (10.3% versus 23.5% for sputa from DS and DR TB subjects, respectively) (Table 2). In comparison, only 1/29 (3.4%) subjects with DS TB and 1/18 (5.6%) of those with DR TB provided sputum with a CFU value higher than the upper bound of the 95% confidence interval of the MPN^−CF^ or MPN^+CF^ value.

**TABLE 2:** Proportion of subjects with drug-sensitive or drug-resistant TB whose sputum was positive for DD-*Mtb* before and after initiation of therapy

Culture filtrate status	Time point^*b*^	% (*n*) of subjects positive for DD-*Mtb* in cohort^*c*^:		*P*^*a*^
		DS	DR	
All	Day 0	20.7 (6/29)	29.4 (5/17)	0.726
	Wk 2	65.2 (15/23)	31.3 (5/16)	0.054
	Mo 2	13.3 (2/15)	0 (0/15)	NA
−CF	Day 0	17.2 (5/29)	22.2 (4/18)	0.716
	Wk 2	65.2 (15/23)	17.6 (3/17)	0.004
	Mo 2	0 (0/15)	0 (0/15)	NA
+CF	Day 0	10.3 (3/29)	23.5 (4/17)	0.397
	Wk 2	60.9 (14/23)	25.0 (4/16)	0.049
	Mo 2	13.3 (2/15)	0 (0/15)	NA

a Comparing DS to DR. NA, not applicable.

b Day 0 was prior to initiation of therapy.

c Positivity was defined as when the CFU value was less than the lower bound of the 95% confidence interval of the MPN value (with or without culture filtrate [CF]). DD, differentially detectable.

**FIG 2:**
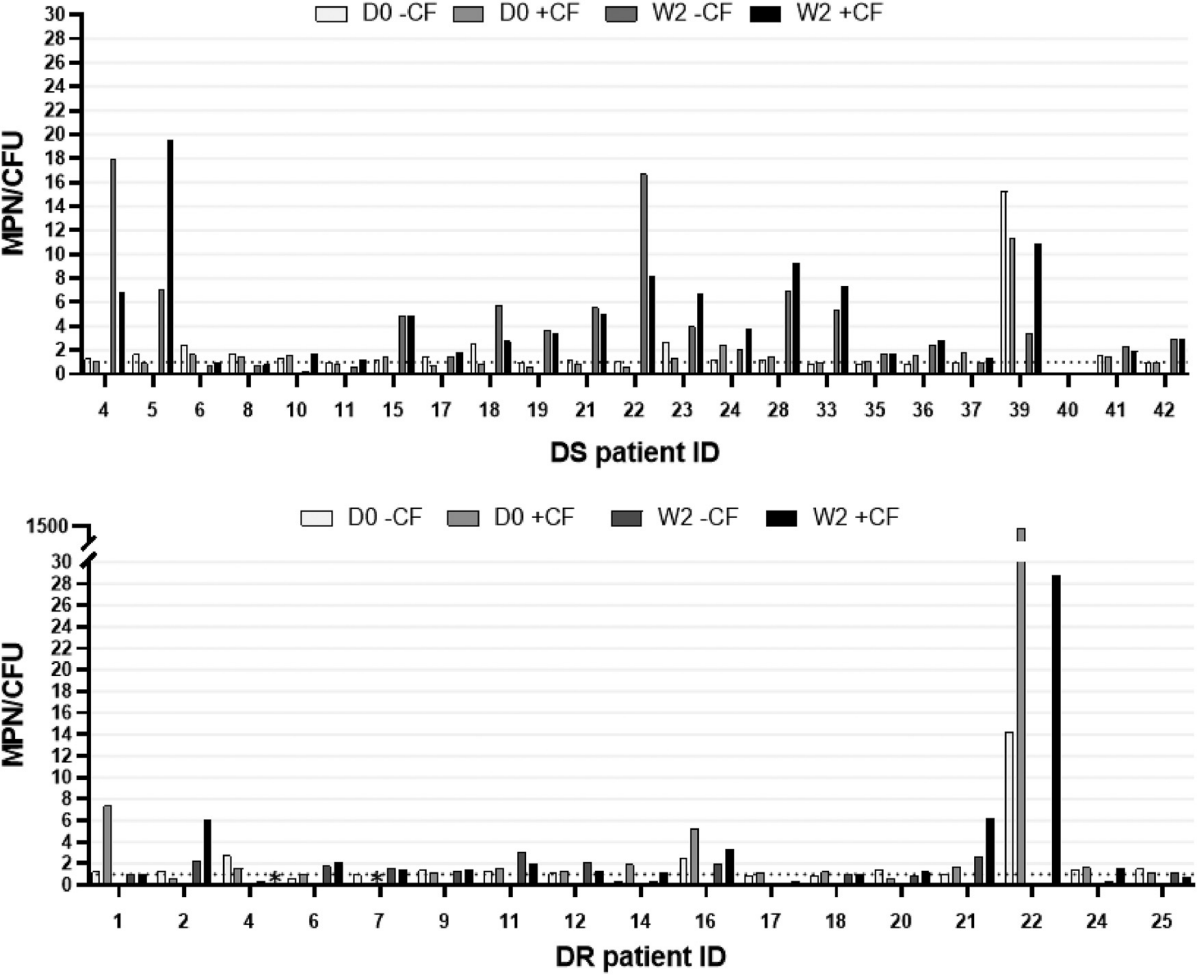
The MPN to CFU ratio (MPN/CFU), with and without culture filtrate (CF), for sputum from subjects with drug-sensitive (DS) and drug-resistant (DR) TB where data for both day 0 (DO) and week 2 (W2) samples were available. The dashed line represents an MPN/CFU ratio of 1. Raw numbers can be found in Table S4. An asterisk (*) indicates data not available due to contamination.

**FIG 3:**
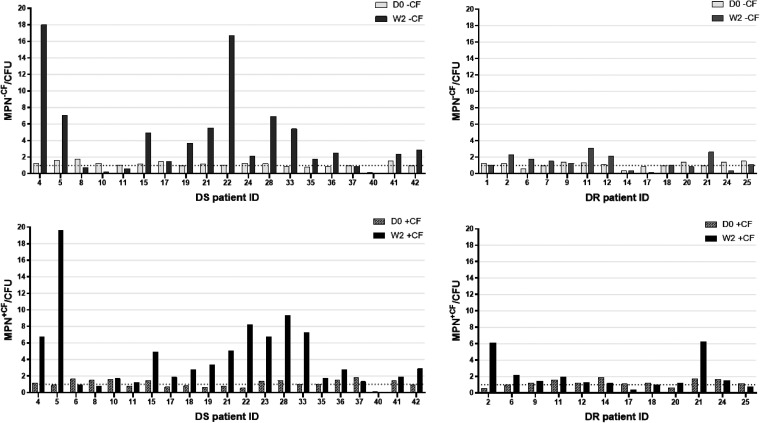
The MPN to CFU ratio (MPN/CFU), with and without culture filtrate (CF), at day 0 (D0) and week 2 (W2) for sputum from subjects with drug-sensitive (DS) and drug-resistant (DR) TB whose sputa showed no DD-*Mtb* at day 0 (D0). The dashed line represents an MPN/CFU ratio of 1. Raw numbers can be found in Table S4.

At 2 weeks post initiation of therapy, the proportion of subjects with DS TB whose sputa were DD-*Mtb* positive increased by about 3-fold, to 65.2% (15/23) and 60.9% (14/23) when the MPN-LD assay was performed without or with CF, respectively (Fig. 1 and Table 2). This, too, is similar to what was observed in an earlier study (69%) that examined a similar cohort of subjects with DS TB at the same study site after 2 weeks of treatment with HRZE (11). Of 5 subjects with DS TB whose sputa were positive for DD-*Mtb* at day 0, 4 (80%) had sputa that remained positive for DD-*Mtb* at week 2 (Fig. 2). Of the subjects with DS TB whose sputa lacked DD-*Mtb* at day 0, approximately 61% showed DD-*Mtb* positivity after 2 weeks of therapy with first-line drugs (Fig. 3 and Table 3).

**TABLE 3:** Proportion of subjects with drug-sensitive or drug-resistant TB whose sputum showed apparent selection or induction of DD-*Mtb* after initiation of treatment^*a*^

Culture filtrate status	% (*n*) of subjects showing selection/induction in cohort:		*P*^*b*^
	DS	DR	
All	61.1 (11/18)	25.0 (3/12)	0.072
−CF	63.2 (12/19)	21.4 (3/14)	0.033
+CF	57.1 (12/21)	16.7 (2/12)	0.033

a Defined as their sputum having no DD-*Mtb* cells at day 0 but showing DD-Mtb at week 2 post initiation of therapy, as determined by comparing MPN with or without culture filtrate (CF) to CFU.

b Comparing DS to DR cohorts.

In contrast, the sputa of subjects with DR TB showed comparable levels of DD-*Mtb* both before (29.4%) and after 2 weeks of therapy with second-line drugs (31.3%) (Fig. 1 and Table 2). This was apparent when the MPN-LD assays were performed both with or without CF (Table 2). Of the four participants in this cohort who had DD-*Mtb*-positive sputa at day 0 and from whom a week 2 sample was also available, only 2 remained positive for DD-*Mtb* at week 2 (Fig. 2). Of the DR TB subjects whose sputa lacked DD-*Mtb* at day 0, only 25% showed DD-*Mtb* positivity after 2 weeks of therapy (Fig. 3 and Table 3).

By month 2, the sputa of 84% (26/31) of all subjects showed no detectable M. tuberculosis growth, and the median count of viable M. tuberculosis in the remaining samples was very low (<20 M. tuberculosis/ml) (see Tables S2 and S3 in the supplemental material). None of the 9 subjects with DS TB whose sputa were positive for DD-*Mtb* at week 2 (including 4 whose sputa were positive for DD-*Mtb* at both day 0 and week 2) showed any M. tuberculosis growth at month 2 (Table S2). In contrast, of the 4 subjects with DR TB whose sputa were DD-*Mtb* positive at week 2, two showed M. tuberculosis growth at month 2 (although neither was positive for DD-*Mtb*) (Table S3). In neither cohort was DD-*Mtb* positivity at day 0 associated with sputum positivity for M. tuberculosis at month 2 (see Table S4 in the supplemental material).

Of the 5 sputa that showed detectable M. tuberculosis growth at 2 months, two were positive for DD-*Mtb*, but only when comparing MPN^+CF^ to CFU, and both belonged to the DS TB cohort. Neither subject’s sputum was positive for DD-*Mtb* at day 0 (week 2 samples were lacking for both). Therefore, the proportion of subjects with DS TB who had DD-*Mtb*-positive sputum fell from 65.2% at week 2 to 13.3% at month 2, and in subjects with DR TB it fell from 31.3% at week 2 to 0% at month 2 (Table 2).

### Relationship of DD-*Mtb* to clinical characteristics and outcomes.

We assessed whether baseline characteristics at enrollment were associated with the occurrence of D*D-Mtb* at either week 0 or week 2 (see Table S5 in the supplemental material). No patient characteristics were associated with the presence of DD-*Mtb* in the sputa of DS TB subjects. HIV positivity (*P* = 0.015), lower hemoglobin (*P* = 0.014), and lower body mass index (BMI; *P* = 0.040) were positively associated with DD-*Mtb* for the cohort with DR TB.

When limiting analyses to presence of DD-*Mtb* only at day 0 (prior to initiation of treatment), no association with patient characteristics was found in the DS cohort. In the DR cohort, HIV positivity (*P* = 0.044) and lack of bilateral disease (*P* = 0.008) were positively associated with DD-*Mtb*. No patient characteristics were associated with the presence of DD-*Mtb* in the sputa of either cohort at week 2 (Table S5).

All 29 DS participants completed 6 months of therapy; 28 achieved either treatment completion or cure, while 1 participant had treatment failure (positive acid-fast bacillus [AFB] smear at month 5) as defined by WHO guidelines. Presence of DD*-Mtb* at month 2 was associated with the only treatment failure of this study (DS no. 12).

Of the 18 participants with DR TB, the median time to culture conversion to negative was 2 months, and mean weight gain at 2 months was 2 kg. The mean time to discharge from the inpatient service was 4 months, and 2 of 18 patients required rehospitalization after initial discharge. There was no significant difference in these parameters between subjects whose sputa were DD-*Mtb* positive versus those who were negative. It is of note that the DR patient with the longest hospitalization (6 months) and persistent symptoms after discharge, which resulted in a rehospitalization, was one of the 5 DR patients with DD-*Mtb* at week 2 (DR no. 21).

### Relative proportions of DD-*Mtb* bacteria in sputum from subjects with DS and DR TB.

For all DD-*Mtb*-positive samples in this study, DD-*Mtb* constituted the majority (>50%) of the total M. tuberculosis bacteria present in the sputum (as defined by an MPN/CFU ratio of >2-fold) (Fig. 1; see also Table S6 in the supplemental material). The median MPN/CFU ratio for DD-*Mtb*-positive samples was 4.4 (interquartile range [IQR], 2.7 to 7.0), meaning that DD-*Mtb* cells comprised a median of nearly 80% of the viable M. tuberculosis cells in the sputa of these participants. When samples from subjects with DS and DR TB were compared, the median MPN/CFU ratios at day 0 were 2.6 and 4.2, respectively; whereas at week 2, the median MPN/CFU ratio increased to 5.0 in sputa from subjects with DS TB but held nearly steady at 3.3 for those with DR TB (Table S6).

Subjects with DS and DR TB showed comparable levels of M. tuberculosis in their sputum prior to initiation of therapy and at 2 weeks post initiation (see Table S7 in the supplemental material). Consistent with this, the rate of decline of M. tuberculosis from day 0 to week 2 was also similar for both cohorts (see Table S8 in the supplemental material). However, as seen in Table S7, at day 0, DD-*Mtb*-positive sputa from the cohort with DR TB showed a 389-fold lower median CFU (*P* = 0.018), a 550-fold lower median MPN^−CF^ (*P* = 0.011), and a 10-fold lower median MPN^+CF^ value (*P* = 0.067) in comparison to those of DD-*Mtb*-positive sputa from the DS cohort. By week 2, these differences were absent. At day 0, DD-*Mtb*-positive sputa from the cohort with DR TB also showed a 1,259-fold lower median CFU (*P* = 0.004), a 550-fold lower median MPN^−CF^ (*P* = 0.007), and a 27-fold lower median MPN^+CF^ value (*P* = 0.011) in comparison to those of DD-*Mtb*-negative sputa from the same cohort. This distinction in levels of viable M. tuberculosis bacteria according to presence or absence of DD-*Mtb* was not seen in sputa from the cohort with DS TB.

### DS- and DR-*Mtb* samples show differing responses to CF.

The median MPN^+CF^/MPN^−CF^ ratio was 1.6-fold higher for sputa from subjects with DR TB than for those with DS TB at day 0 (*P* = 0.032). This ratio was 3.2-fold when the comparison was limited to DD-*Mtb*-positive samples (Table S7). When the comparison was made between MPN^+CF^ and MPN^−CF^ values for individual sputa that contained DD-*Mtb*, the MPN^+CF^ assay detected a 2.3-fold higher median DD-*Mtb* number for samples from subjects with DR TB but a 1.5-fold lower median DD-*Mtb* number for samples from subjects with DS TB. In other words, on average, CF appeared to promote the growth of DD-*Mtb* bacteria from the DR cohort while inhibiting the growth of DD-*Mtb* bacteria in sputa from the DS cohort at day 0.

## DISCUSSION

Here, we report that subjects with DS and DR TB show differing incidence of DD-*Mtb* in their sputa after initiation of therapy. We found that similar proportions of subjects with DS and DR TB—20.7% and 29.4%, respectively—presented with sputum positive for DD-*Mtb* prior to therapy. These proportions resemble what was reported earlier (21%) for subjects with DS TB by a different experimental investigator applying the same methods at the same study site (11). After 2 weeks of HRZE, the proportion of subjects whose sputa were DD-*Mtb*-positive more than tripled, again mirroring earlier observations (11). In contrast, the proportion of subjects with DR TB whose sputa were DD-*Mtb* positive changed little after 2 weeks on a multidrug regimen that included pyrazinamide but lacked rifampin, isoniazid, and ethambutol. These observations are consistent with the hypotheses that HRZE or one of its components, perhaps rifampin (8), promotes an increased proportion of DD-*Mtb* bacteria in the sputum by selective elimination of M. tuberculosis bacteria that are not DD-*Mtb* or by induction of DD-*Mtb* (and second-line therapy does not do so), or that the more extensive disease or prior treatment in most subjects receiving second-line therapy (along with any potential accompanying resistance-associated mutations) limits selection or induction of DD-*Mtb*.

Studies of DD-*Mtb* cells generated *in vitro* reveal that they are bacteria that have sustained oxidative damage at a sublethal level and can regain the capacity to form colonies on agar if their replication is delayed until they undergo repair (Saito et al., unpublished). While disengaged from replication, DD-*Mtb* are profoundly antibiotic resistant (8, 14, 15). While intermediate levels of oxidative damage give rise to DD-*Mtb*, more extensive oxidative damage kills them (Saito et al., unpublished). Perhaps the exposure of subjects with DR TB to oxidant-generating drugs like clofazimine (18) damages a higher proportion of their M. tuberculosis bacteria beyond the point of repair.

The notion that sublethal damage can promote formation of DD-*Mtb* cells is consistent with the finding that HIV-positive (HIV+) subjects with TB who had peripheral blood CD4 counts of >200 cells/ml were more likely to have DD-*Mtb* in their sputum than those with CD4 counts of <200 cells/ml (10). In the present study, subjects with DS TB who had DD-*Mtb*-positive sputum at day 0 had a 3.5-fold lower median CFU count in their sputum than those whose sputum lacked DD-*Mtb* (see Table S7 in the supplemental material). Perhaps there is a level of host immunity that suffices to reduce but not eliminate M. tuberculosis and inflicts the intermediate level of damage that favors formation of DD-*Mtb*. Similarly, subjects with DR TB who had DD-*Mtb*-positive sputum at day 0 had a >1,000-fold lower median CFU count in their sputum than that of their DD-*Mtb*-negative counterparts, perhaps highlighting the additive effects of host immunity and previous exposure to first-line therapies. Consistent with this, Xpert positivity indicative of very low or low M. tuberculosis counts in sputum was positively associated with the presence of DD-*Mtb* (*P* = 0.014) (see Table S5 in the supplemental material).

DD-*Mtb* populations in the sputum did not persist beyond the first 2 months of therapy for most subjects. Specifically, 9/9 subjects with DS TB and 7/7 with DR TB whose sputum previously contained DD-*Mtb* bacteria lacked evidence of DD-*Mtb* at 2 months. Nonetheless, DD-*Mtb* was detected at month 2 in the sputa of 2 subjects with DS TB whose sputa did not show DD-*Mtb* at day 0 (week 2 samples were missing for both). More generally, sputa from most subjects showed no evidence of viable M. tuberculosis cells at 2 months by any of the assays used, and when M. tuberculosis was detected at 2 months, the levels were very low. These findings are consistent with a report that found no M. tuberculosis growth after 3 months of treatment in the sputum of 17 subjects with DS TB using assays similar to those employed here (5). Therefore, detection of DD-*Mtb* at an earlier time point did not foretell sputum positivity by any assay used at 2 months of treatment.

Nonetheless, those subjects whose sputum contains detectable DD-*Mtb* at some point before or during their treatment may face a more challenging clinical course than those whose sputum does not. The two subjects who responded most poorly to treatment in each cohort, namely, the subject with DS TB whose treatment failed and the subject with DR TB who required rehospitalization, both had DD-*Mtb* detected during treatment. However, the number of subjects who did poorly was too small for statistical assessment, highlighting a limitation of the current study. Larger studies with longer follow-up times will be needed to draw firm conclusions about the role of DD-*Mtb* in treatment outcome. Beltran et al. (6) identified DD-*Mtb* in the sputum of two subjects with DS TB who subsequently relapsed, while Almeida Júnior et al. (5) found no association between DD-*Mtb* and treatment failure or relapse during the first 6 months of treatment for DS TB. While CF had a variable effect on M. tuberculosis growth in this and previous studies (10–12, 17), for both subjects for whom DD-*Mtb* was associated with poorer clinical outcome, CF markedly improved M. tuberculosis recovery in the MPN-LD assay, similarly to what Beltran et al. (6) observed in their subjects who went on to relapse.

Additionally, CF tended to promote recovery of DD-*Mtb* in sputum from subjects with DR TB but tended to suppress recovery of DD-*Mtb* in sputum from subjects with DS TB. The latter effect was alleviated in sputa from the cohort with DS TB after initiation of drug treatment, suggesting that the prior drug exposure experienced by subjects with DR TB may have contributed to this difference. What components of CF exert these effects and by what mechanisms remain unknown (12), making it difficult to draw biologic inferences. As a practical matter, it is advisable to perform MPN-LD assays both with and without CF to maximize detection of DD-*Mtb*.

In the future, a rapid, quantitative, and culture-independent means of estimating the number of DD-*Mtb* cells in clinical specimens could help address the many open questions that remain regarding DD-*Mtb*. Given that culture conversion is already known to be a poor predictor of TB cure, there is a need for the development of diagnostics that more accurately reflect total viable M. tuberculosis populations in sputa, which can be used to screen investigational anti-TB drugs and identify individuals who qualify for shorter treatments, with the aim of ultimately improving TB outcomes.

## MATERIALS AND METHODS

### Study design and patient populations.

This prospective observational study was performed at the Groupe Haïtien d’Étude du Sarcome de Kaposi et des Infectieuses Opportuniste (GHESKIO) centers in Port au Prince, Haiti, and approved by the institutional review boards of both GHESKIO and Weill Cornell Medicine. All participants provided written informed consent and scored at least 90% on an assessment of understanding quiz before enrollment. HIV+ subjects from both cohorts were on antiretroviral therapy at the time of enrollment and remained on treatment during the period of the study.

The DS cohort met the following criteria: the participants were ≥ 18 years of age; had been diagnosed as having active pulmonary TB based on clinical symptoms and signs, chest radiography, and Xpert Mtb/RIF assay (Cepheid, Sunnyvale, CA) positivity, without indication of rifampin resistance; were free of extrapulmonary manifestations of TB or concomitant illnesses that might interfere with treatment in the opinion of the participant’s care provider; had no history of TB or its treatment; and were planning to initiate treatment at the GHESKIO clinic. The DR cohort met the same criteria, except that the Xpert test indicated rifampin resistance and participants were not excluded if they had previously been treated for drug-sensitive TB. Sputum was collected from both cohorts prior to initiation of therapy, and at 2 weeks and 2 months after initiation of therapy. TB symptoms (cough, dyspnea, hemoptysis, fever, and pleuritic chest pain) were graded according to the Division of AIDS (DAIDS) grading system (19).

Participants with DS TB were followed in the GHESKIO outpatient clinic for the duration of their directly observed therapy (DOT). Participants received isoniazid (H), rifampin (R), ethambutol (E), and pyrazinamide (Z) for 2 months, then HR for 4 months. During the first 2 months, participants had clinical visits every 2 weeks at which time signs and symptoms were recorded, and then every month for the remainder of their treatment regimen. Treatment outcome was determined based on Haitian and WHO guidelines—spot sputum samples were collected during clinical visits for AFB smear at months 2, 5, and 6 and underwent culture only if AFB positive. Subjects also provided overnight sputum samples on day 0 and at week 2 and month 2 for the laboratory analyses performed in this study. One month after completion of therapy, participants had a final clinical visit and were counseled to return if their condition worsened. Participants were not actively followed after completion of treatment, and so the relationship with posttreatment recurrence could not be assessed.

Participants with DR TB were hospitalized in GHESKIO’s inpatient multidrug-resistant (MDR)-TB hospital for approximately the first 4 months of treatment. They were discharged once they demonstrated clinical improvement and negative M. tuberculosis cultures and had designated family support to ensure treatment adherence upon discharge. Treatment was in accordance with Haitian and WHO guidelines, with DOT regimens comprised of bedaquiline, levofloxacin, linezolid, clofazimine, and pyrazinamide. Bedaquiline was discontinued after 6 months and linezolid after 12 months, with the remaining drugs continued to complete 20 months of therapy. Once discharged, participants were followed in GHESKIO’s outpatient MDR-TB clinics with monthly outpatient follow-up. Spot sputum samples were provided monthly for AFB smear and monthly for culture during months 1 to 8, every other month during months 9 to 18, and then monthly for the final 3 months of treatment. For the current study, we documented the following outcomes: length of hospitalization and any readmission, change in weight by month 2, persistent TB symptoms, and time to culture conversion.

### Sputum processing and microbiological assays.

Subjects collected overnight sputum into a cool box with ice packs (4°C) from 5 p.m. to 9 a.m. Samples reached the GHESKIO laboratory the same morning, with >79% of samples being processed the same day, >92% being processed within 2 days, and all being processed within 5 days. Experimental work took place in a biosafety level 3 laboratory with appropriate safety guidelines and personal protective equipment.

Decontamination of sputum, preparation of culture filtrate (CF), and CFU and MPN-LD assays were conducted as reported previously (11). Decontamination was monitored by plating aliquots on blood agar. Results of LD assays performed without CF are referred to as MPN^−CF^ and those with CF as MPN^+CF^. CFU and MPN plates were placed inside partially sealed Ziploc bags and placed inside a 37°C incubator (unhumidified and without CO_2_) for up to 9 weeks, with reads performed after 3 weeks and then every 2 weeks thereafter until no additional growth was observed between two consecutive reads. Positive colonies were identified visually, and any suspect positive wells were confirmed for presence of M. tuberculosis by using an SD Bioline TB AG MPT64 rapid antigen test (only for MPN^−CF^ plates). Contamination was ruled out by plating on blood agar.

### Data analysis.

Data were uploaded to REDCap (20, 21), a secure web-based software platform designed to support data capture for research studies. A second investigator used the raw data files to repeat and verify the calculations. Confidence intervals (95%) were calculated separately for assays with and without CF from the results of 10 independent dilution series per sample (22). These were compared to the average CFU values on two agar plates for a sample taken from each dilution of each LD series. DD-*Mtb* was recorded as present when the number of CFU calculated for the original sample was less than the lower 95% confidence interval of the number of viable M. tuberculosis bacteria in the original sample calculated by the MPN method based on growth of M. tuberculosis in the LD series. CFU and MPN-LD assays had a lower limit of detection (LLD) of 3 M. tuberculosis CFU/ml. In instances of contamination of CFU and/or MPN-LD plates, the LLD increased proportionately. If a sample showed no M. tuberculosis growth, the next whole number below the LLD was used for the purposes of MPN/CFU ratios. Samples were assigned an MPN/CFU ratio of 1 if <10 M. tuberculosis/ml were detected in all three assay formats (CFU, MPN^−CF^, and MPN^+CF^).

Numbers of viable M. tuberculosis are presented as log_10_ values per ml of sputum. The ratio of DD-*Mtb* to CFU in a sample is calculated by subtracting the log_10_ values for CFU assays from the log_10_ value for MPN-LD assays and determining the antilog of the difference, which is presented as a whole number. Results at day 0 and week 2 were compared only for subjects whose data were available at both time points. The Wilcoxon signed rank test (a nonparametric version of the paired *t* test) was used to compare two quantitative results at one time point and the change in one quantitative result with time. The Wilcoxon rank sum test (a nonparametric version of a two-sample *t* test) was used to compare distribution of continuous features between samples from subjects with DS versus DR TB. Fisher’s exact test was used to assess the association between two categorical features (presence of DD-*Mtb* and DS/DR status of the M. tuberculosis at the outset). All tests were performed for two-tailed hypotheses, and *P* values of <0.05 were considered statistically significant. Statistical analyses were performed using R v4.0.2.
