# Supplementary material for: Characterization of Differentially Detectable Mycobacterium tuberculosis in the Sputum of Subjects with Drug-Sensitive or Drug-Resistant Tuberculosis before and after Two Months of Therapy
Source: Antimicrob Agents Chemother. 2021 Jul 16;65(8):e00608-21. doi: 10.1128/AAC.00608-21 (PMC8284451; doi:10.1128/AAC.00608-21)
Supplement: Supplemental file 1 — Supplemental Tables S1 to S3 and S5 to S8. Download AAC00608-21_Supp_1_seq9.pdf, PDF file, 0.8 MB [file aac00608-21_supp_1_seq9.pdf]

Table S1. Results of drug susceptibility testing of Mtb isolated from study participants with DR TB who were enrolled in this study (n=18).

DR subject #	Rifampin	Isoniazid	Ethambutol	Pyrazinamide	Streptomycin	Capreomycin	Cycloserine	Ethionamide	Kanamycin	Para-aminocylic acid	Amikacin	Ofloxacin	Rifabutin
**1**	R	S	S	S	S	S	S	S	S	S	S	S	R
**2**	R	R	R	R	R	S	S	S	S	S	S	S	R
**4**	R	R	R	S	R	S	S	S	S	S	S	S	S
**6**	R	R	R	S	S	S	S	S	S	S	S	S	R
**7**	R	R	R	R	S	S	S	R	S	S	S	S	R
**9**	R	R	R	R	R	S	S	S	S	S	S	S	I
**11**	R	R	R	S	S	S	S	S	S	S	S	S	R
**12**	R	R	R	R	S	S	S	S	S	S	S	S	R
**14**	R	R	S	S	S	S	S	S	S	S	S	S	R
**15**	R	R	R	S	S	S	S	S	S	S	S	S	R
**16**	R	R	R	R	S	S	S	S	S	S	S	S	R
**17**	R	R	R	R	S	S	S	S	S	S	S	S	R
**18**	R	R	R	S	S	S	S	S	S	S	S	S	S
**20**	R	R	R	S	R	S	S	S	S	S	S	S	R
**21**	R	R	R	S	R	S	S	S	S	S	S	S	R
**22**	R	R	S	S	S	S	S	S	S	S	S	S	R
**24**	R	R	R	S	S	S	S	S	S	S	S	S	R
**25**	R	R	R	R	S	S	S	S	S	S	S	S	S

R, resistant; S, sensitive; I, indeterminate.

Table S2. Quantification of Mtb in sputum from subjects with drug sensitive (DS) TB at 2 months post-initiation of therapy based on CFU, MPN^{-CF}, and MPN^{+CF} assays. Subjects whose sputum was positive for Mtb growth have been bolded. DS subjects 12 and 13 were positive for DD Mtb at month 2.

	Month 2				
**DS subject ID:**	**CFU Mtb/ml [LLD]**	**MPN^{-CF} Mtb/ml [LLD] (95% CI)**	**MPN^{+CF} Mtb/ml [LLD] (95% CI)**	**DD Mtb at Day 0?**	**DD Mtb at Week2?**
11	0 [3]	0 [3] (0-12)	0 [3] (0-12)		
**12**	**47 [3]**	**27 [3] (12-63)**	**112[1] [3] (52-252)**		**N/A**
**13**	**47 [3]**	**38 [3] (16-91)**	**273[1] [3] (84-840)**		**N/A**
15	0 [6]	0 [3] (0-12)	0 [3] (0-13)		Yes (-CF/+CF)
18	0 [3]	0 [3] (0-12)	0 [3] (0-12)	Yes (-CF)	Yes (-CF/+CF)
22	0 [3]	0 [3] (0-12)	0 [3] 0-12)		Yes (-CF/+CF)
23	0 [25]	Contam	Contam	Yes (-CF)	Yes (-CF/+CF)
24	0 [3]	0 [3] (0-12)	0 [3] (0-12)	Yes (+CF)	Yes (-CF/+CF)
28	0 [3]	0 [3] (0-12)	0 [3] (0-12)		Yes (-CF/+CF)
30	0 [3]	0 [3] (0-12)	0 [3] (0-12)		N/A
31	Contam	0 [3] (0-12)	0 [4] (0-17)		N/A
33	0 [3]	0 [3] (0-12)	0 [3] (0-12)		Yes (-CF/+CF)
35	0 [13]	0 [3] (0-12)	0 [4] (0-16)		
36	0 [3]	0 [4] (0-14)	0 [3] (0-13)		Yes (-CF/+CF)
37	0 [4]	0 [3] (0-13)	0 [3] (0-13)		
39	0 [3]	0 [3] (0-12)	0 [3] (0-13)	Yes (-CF/+CF)	Yes (-CF/+CF)

[1] Positive for DD Mtb by MPN^{+CF}/CFU; LLD, lower limit of detection; CI, confidence interval; Contam, contaminated; N/A, not available; CF, culture filtrate

Table S3. Quantification of Mtb in sputum from subjects with drug resistant (DR) TB at 2 months post-initiation of therapy based on CFU, MPN^{-CF}, and MPN^{+CF} assays. Subjects whose sputum was positive for Mtb growth have been bolded. None was positive for DD Mtb at month 2.

	**Month 2**				
**DR subject ID:**	**CFU Mtb/ml [LLD]**	**MPN^{-CF} Mtb/ml [LLD] (95% CI)**	**MPN^{+CF} Mtb/ml [LLD] (95% CI)**	**DD Mtb at Day 0?**	**DD Mtb at Week2?**
1	0<br>[3]	0<br>[3] (0-12)	0<br>[3] (0-12)	Yes<br>(+CF)	
**2**	**16**<br>**[3]**	**21**<br>**[3] (8-52)**	**3**<br>**[3] (0-25)**		**Yes**<br>**(-CF/+CF)**
4	0<br>[6]	0<br>[3] (0-12)	0<br>[3] (0-12)	Yes<br>(-CF)	N/A
6	0<br>[3]	0<br>[3] (0-12)	0<br>[3] (0-12)		
7	0<br>[33]	0<br>[4] (0-14)	0<br>[4] (0-14)		
9	0<br>[6]	0<br>[3] (0-12)	0<br>[3] (0-12)		
11	0<br>[6]	0<br>[3] (0-12)	0<br>[3] (0-12)		Yes<br>(-CF)
15	0<br>[6]	0<br>[3] (0-12)	0<br>[3] (0-12)	Yes<br>(-CF/+CF)	N/A
16	0<br>[3]	0<br>[3] (0-12)	0<br>[3] (0-12)	Yes<br>(-CF/+CF)	Yes<br>(+CF)
17	0<br>[3]	0<br>[3] (0-12)	0<br>[3] (0-12)		
18	0<br>[3]	0<br>[3] (0-13)	0<br>[4] (0-13)		
**20**	**5**<br>**[3]**	**0**<br>**[3] (0-12)**	**3**<br>**[3] (0-25)**		
**21**	**3**<br>**[3]**	**0**<br>**[3] (0-12)**	**7**<br>**[3] (2-29)**		**Yes**<br>**(-CF/+CF)**
**24**	0<br>[3]	0<br>[3] (0-12)	0<br>[3] (0-12)		
**25**	0<br>[3]	0<br>[3] (0-12)	0<br>[3] (0-12)		

LLD, lower limit of detection; CI, confidence interval; CF, culture filtrate CF, culture filtrate

Table S4. Table summarizing patient characteristics at time of enrollment combined with Mtb counts obtained with CFU, MPN-LD without CF (MPN^{-CF}) and with CF (MPN^{+CF}) for each participant at day 0, week 2, and month 2.

Table S5. Statistical analysis of patient characteristics at time of enrollment and their association with presence of DD Mtb in sputum of subjects with drug sensitive (DS) and/or drug resistant (DR) TB at day 0 and week 2.

	DS (day 0)		
	**DD Mtb -**	**DD Mtb +**	**p-value**
n	23	6	
Male, n (%)	15 (65.2)	3 (50.0)	0.646
HIV+, n (%)	1 (4.3)	0 (0.0)	1.000
Cavities, n (%)	11 (47.8)	3 (50.0)	1.000
Bilateral disease, n (%)	8 (34.8)	1 (16.7)	0.633
No prior treatment, n (%)	23 (100.0)	6 (100.0)	NA
No blood tinged sputum, n (%)	23 (100.0)	6 (100.0)	NA
Pleuritic chest pain, n (%)	7 (30.4)	3 (50.0)	0.633
Fever, n (%)	10 (43.5)	0 (0.0)	0.068
Age (median [IQR])	34.0 [28.5, 48.0]	32.0 [26.5, 33.7]	0.257
Hemoglobin (median [IQR])	10.8 [10.2, 11.5]	10.2 [9.45, 10.8]	0.246
Creatinine (median [IQR])	0.70 [0.60, 0.80]	0.65 [0.60, 0.70]	0.408
Pulse oximetry (median [IQR])	97.0 [95.2, 98.0]	96.5 [95.2, 97.7]	0.629
BMI (median [IQR])	18.8 [16.9, 20.8]	19.7 [19.1, 22.1]	0.216
Dyspnea, n (%)	14 (60.9)	2 (33.3)	0.364
Cough, n (%)	23 (100.0)	6 (100.0)	NA
Xpert Medium/High, n (%)	20 (87.0)	4 (66.7)	0.269

	DS (week 2)		
	DD Mtb -	DD Mtb +	p-value
n	8	15	
Male, n (%)	6 (75.0)	9 (60.0)	0.657
HIV+, n (%)	1 (12.5)	0 (0.0)	0.348
Cavities, n (%)	3 (37.5)	8 (53.3)	0.667
Bilateral disease, n (%)	2 (25.0)	6 (40.0)	0.657
No prior treatment, n (%)	8 (100.0)	15 (100.0)	NA
No blood tinged sputum, n (%)	8 (100.0)	15 (100.0)	NA
Pleuritic chest pain, n (%)	5 (62.5)	5 (33.3)	0.221
Fever, n (%)	4 (50.0)	4 (26.7)	0.371
Age (median [IQR])	30.0 [28.7, 37.2]	34.0 [26.5, 41.5]	0.746
Hemoglobin (median [IQR])	11.0 [10.6, 11.4]	10.7 [9.8, 11.3]	0.627
Creatinine (median [IQR])	0.65 [0.60, 0.90]	0.70 [0.60, 0.75]	0.641
Pulse oximetry (median [IQR])	98.0 [97.5, 98.5]	97.0 [96.5, 98.0]	0.247
BMI (median [IQR])	18.9 [17.5, 19.3]	19.0 [16.9, 21.0]	0.747
Dyspnea, n (%)	4 (50.0)	8 (53.3)	1.000
Cough, n (%)	8 (100.0)	15 (100.0)	NA
Xpert Medium/High, n (%)	7 (87.5)	11 (73.3)	0.621

	DS (day 0 & week 2)		
	**DD Mtb -**	**DD Mtb +**	**p-value**
n	7	16	
Male, n (%)	5 (71.4)	10 (62.5)	1.000
HIV+, n (%)	1 (14.3)	0 (0.0)	0.304
Cavities, n (%)	3 (42.9)	8 (50.0)	1.000
Bilateral disease, n (%)	1 (14.3)	7 (43.8)	0.345
No prior treatment, n (%)	7 (100.0)	16 (100.0)	NA
No blood tinged sputum, n (%)	7 (100.0)	16 (100.0)	NA
Pleuritic chest pain, n (%)	4 (57.1)	6 (37.5)	0.650
Fever, n (%)	4 (57.1)	4 (25.0)	0.182
Age (median [IQR])	30.0 [28.5, 40.0]	34.0 [26.7, 39.2]	0.789
Hemoglobin (median [IQR])	11.2 [10.5, 11.4]	10.7 [10.0, 11.2]	0.663
Creatinine (median [IQR])	0.60 [0.60, 0.80]	0.70 [0.60, 0.80]	0.918
Pulse oximetry (median [IQR])	98.0 [97.2, 98.0]	97.0 [96.7, 98.2]	0.570
BMI (median [IQR])	18.8 [17.5, 19.4]	19.1 [17.0, 20.9]	0.593
Dyspnea, n (%)	4 (57.1)	8 (50.0)	1.000
Cough, n (%)	7 (100.0)	16 (100.0)	NA
Xpert Medium/High, n (%)	7 (100.0)	11 (68.8)	0.272

	DR (day 0)		
	DD Mtb -	DD Mtb +	p-value
n	13	5	
Male, n (%)	7 (53.8)	2 (40.0)	1.000
HIV+, n (%)	1 (7.7)	3 (60.0)	0.044
Cavities, n (%)	6 (46.2)	2 (40.0)	1.000
Bilateral disease, n (%)	12 (92.3)	1 (20.0)	0.008
Received prior TB treatment, n (%)	10 (76.9)	5 (100.0)	0.522
Blood tinged sputum, n (%)	2 (15.4)	0 (0.0)	1.000
Pleuritic chest pain, n (%)	7 (53.8)	3 (60.0)	1.000
Fever, n (%)	2 (15.4)	0 (0.0)	1.000
Age (median [IQR])	31.0 [28.0, 43.0]	33.0 [29.0, 37.0]	0.882
Hemoglobin (median [IQR])	11.2 [10.3, 12.1]	10.3 [10.3, 10.6]	0.103
Creatinine (median [IQR])	0.70 [0.60, 0.70]	0.50 [0.40, 0.60]	0.130
Pulse oximetry (median [IQR])	97.0 [97.0, 98.0]	99.0 [98.0, 99.0]	0.239
BMI (median [IQR])	17.9 [16.7, 20.2]	17.5 [17.0, 18.6]	0.657
Dyspnea, n (%)	11 (84.6)	4 (80.0)	1.000
Cough, n (%)	12 (92.3)	5 (100.0)	1.000
Xpert Medium/High, n (%)	12 (92.3)	4 (80.0)	0.490

	DR (week 2)		
	DD Mtb -	DD Mtb +	p-value
n	12	5	
Male, n (%)	6 (50.0)	3 (60.0)	1.000
HIV+, n (%)	2 (16.7)	2 (40.0)	0.538
Cavities, n (%)	6 (50.0)	2 (40.0)	1.000
Bilateral disease, n (%)	9 (75.0)	4 (80.0)	1.000
Received prior TB treatment, n (%)	10 (83.3)	4 (80.0)	1.000
Blood tinged sputum, n (%)	1 (8.3)	1 (20.0)	0.515
Pleuritic chest pain, n (%)	6 (50.0)	3 (60.0)	1.000
Fever, n (%)	1 (8.3)	1 (20.0)	0.515
Age (median [IQR])	30.0 [27.7, 34.0]	42.0 [37.0, 43.0]	0.113
Hemoglobin (median [IQR])	11.3 [10.7, 12.1]	10.3 [10.3, 10.3]	0.072
Creatinine (median [IQR])	0.65 [0.60, 0.75]	0.50 [0.40, 0.70]	0.159
pulse oximetry (median [IQR])	97.5 [97.0, 98.2]	98.0 [95.0, 99.0]	0.957
BMI (median [IQR])	18.5 [17.1, 20.9]	17.0 [16.5, 17.1]	0.058
Dyspnea, n (%)	9 (75.0)	5 (100.0)	0.515
Cough, n (%)	11 (91.7)	5 (100.0)	1.000
Xpert Medium/High, n (%)	12 (100.0)	3 (60.0)	0.074

	DR (day 0 & week 2)		
	**DD Mtb -**	**DD Mtb +**	**p-value**
n	10	7	
Male, n (%)	4 (40.0)	5 (71.4)	0.335
HIV+, n (%)	0 (0.0)	4 (57.1)	0.015
Cavities, n (%)	5 (50.0)	3 (42.9)	1.000
Bilateral disease, n (%)	9 (90.0)	4 (57.1)	0.250
Received prior TB treatment, n (%)	8 (80.0)	6 (85.7)	1.000
Blood tinged sputum, n (%)	1 (10.0)	1 (14.3)	1.000
Pleuritic chest pain, n (%)	5 (50.0)	4 (57.1)	1.000
Fever, n (%)	1 (10.0)	1 (14.3)	1.000
Age (median [IQR])	29.5 [27.2, 36.0]	37.0 [31.0, 42.5]	0.203
Hemoglobin (median [IQR])	11.4 [11.2, 12.2]	10.3 [9.9, 10.6]	0.014
Creatinine (median [IQR])	0.65 [0.60, 0.70]	0.60 [0.45, 0.70]	0.367
Pulse oximetry (median [IQR])	97.0 [97.0, 98.0]	98.0 [96.5, 99.0]	0.480
BMI (median [IQR])	19.0 [17.3, 22.2]	17.0 [16.0, 17.3]	0.040
Dyspnea, n (%)	8 (80.0)	6 (85.7)	1.000
Cough, n (%)	9 (90.0)	7 (100.0)	1.000
Xpert Medium/High, n (%)	10 (100.0)	5 (71.4)	0.154

	DS & DR (day 0)		
	DD Mtb -	DD Mtb +	p-value
n	36	11	
Male, n (%)	22 (61.1)	5 (45.5)	0.489
HIV+, n (%)	2 (5.6)	3 (27.3)	0.076
Cavities, n (%)	17 (47.2)	5 (45.5)	1.000
Bilateral disease, n (%)	20 (55.6)	2 (18.2)	0.041
Received prior TB treatment, n (%)	10 (27.8)	5 (45.5)	0.292
Blood tinged sputum, n (%)	2 (5.6)	0 (0.0)	1.000
Pleuritic chest pain, n (%)	14 (38.9)	6 (54.5)	0.489
Fever, n (%)	12 (33.3)	0 (0.0)	0.044
Age (median [IQR])	32.5 [28.0, 46.5]	33.0 [27.0, 34.0]	0.359
Hemoglobin (median [IQR])	11.1 [10.3, 11.6]	10.3 [9.7, 10.8]	0.048
Creatinine (median [IQR])	0.70 [0.60, 0.80]	0.60 [0.50, 0.70]	0.108
Pulse oximetry (median [IQR])	97.0 [96.0, 98.0]	98.0 [95.5, 99.0]	0.664
BMI (median [IQR])	18.2 [16.8, 20.8]	19.1 [17.3, 19.9]	0.744
Dyspnea, n (%)	25 (69.4)	6 (54.5)	0.472
Cough, n (%)	35 (97.2)	11 (100.0)	1.000
Xpert Medium/High, n (%)	32 (88.9)	8 (72.7)	0.330

	DS & DR (week 2)		
	DD Mtb -	DD Mtb +	p-value
n	20	20	
Male, n (%)	12 (60.0)	12 (60.0)	1.000
HIV+, n (%)	3 (15.0)	2 (10.0)	1.000
Cavities, n (%)	9 (45.0)	10 (50.0)	1.000
Bilateral disease, n (%)	11 (55.0)	10 (50.0)	1.000
Received prior TB treatment, n (%)	10 (50.0)	4 (20.0)	0.096
Blood tinged sputum, n (%)	1 (5.0)	1 (5.0)	1.000
Pleuritic chest pain, n (%)	11 (55.0)	8 (40.0)	0.527
Fever, n (%)	5 (25.0)	5 (25.0)	1.000
Age (median [IQR])	30.0 [28.0, 34.0]	35.0 [28.5, 43.7]	0.273
Hemoglobin (median [IQR])	11.2 [10.6, 11.6]	10.5 [10.0, 11.1]	0.074
Creatinine (median [IQR])	0.65 [0.60, 0.90]	0.70 [0.58, 0.70]	0.403
Pulse oximetry (median [IQR])	98.0 [97.0, 98.5]	97.0 [95.7, 98.2]	0.304
BMI (median [IQR])	18.7 [17.4, 19.9]	17.6 [16.7, 20.4]	0.465
Dyspnea, n (%)	13 (65.0)	13 (65.0)	1.000
Cough, n (%)	19 (95.0)	20 (100.0)	1.000
Xpert Medium/High, n (%)	19 (95.0)	14 (70.0)	0.091

	DS & DR (day 0 & week 2)		
	**DD Mtb -**	**DD Mtb +**	**p-value**
n	17	23	
Male, n (%)	9 (52.9)	15 (65.2)	0.522
HIV+, n (%)	1 (5.9)	4 (17.4)	0.373
Cavities, n (%)	8 (47.1)	11 (47.8)	1.000
Bilateral disease, n (%)	10 (58.8)	11 (47.8)	0.538
Received prior TB treatment, n (%)	8 (47.1)	6 (26.1)	0.198
Blood tinged sputum, n (%)	1 (5.9)	1 (4.3)	1.000
Pleuritic chest pain, n (%)	9 (52.9)	10 (43.5)	0.750
Fever, n (%)	5 (29.4)	5 (21.7)	0.717
Age (median [IQR])	30.0 [28.0, 37.0]	34.0 [29.0, 42.5]	0.366
Hemoglobin (median [IQR])	11.3 [10.7, 11.6]	10.7 [9.9, 11.0]	0.029
Creatinine (median [IQR])	0.60 [0.60, 0.70]	0.70 [0.60, 0.75]	0.811
Pulse oximetry (median [IQR])	97.5 [97.0, 98.0]	98.0 [96.5, 99.0]	0.953
BMI (median [IQR])	18.8 [17.5, 20.2]	17.8 [16.6, 19.7]	0.331
Dyspnea, n (%)	12 (70.6)	14 (60.9)	0.739
Cough, n (%)	16 (94.1)	23 (100.0)	0.425
Xpert Medium/High, n (%)	17 (100.0)	16 (69.6)	0.014

Table S6. Proportionate representation of DD Mtb in sputum as measured by the ratio of MPN with or without culture filtrate (CF)/CFU for all sputum samples, DD Mtb positive sputum samples, or DD Mtb negative sputum samples.

			**Day 0**		**Week 2**	
			**DS**	**DR**	**DS**	**DR**
**All samples**		**MPN^{-CF}/CFU**<br>median [IQR]	1.2<br>[0.95-1.6]<br>n=28	1.3<br>[0.94-1.5]<br>n=18	2.9[2]<br>[1.2-5.5]<br>n=23	1.1[2]<br>[0.36-1.9]<br>n=17
		**MPN^{+CF}/CFU**<br>median [IQR]	1.1[1]<br>[0.81-1.5]<br>n=28	1.6[1]<br>[1.2-1.9]<br>n=17	3.2<br>[1.7-6.7]<br>n=23	1.4<br>[1.0-2.4]<br>n=16
**DD Mtb positive samples**	**All**	**MPN/CFU**<br>median [IQR]	2.6<br>[2.4-5.4]<br>n=8	4.2<br>[2.4-9.1]<br>n=8	5.0<br>[3.4-7.0]<br>n=29	3.3<br>[2.8-6.2]<br>n=7
	**-CF**	**MPN^{-CF}/CFU**<br>median [IQR]	2.6<br>[2.5-3.5]<br>n=5	2.6<br>[2.4-5.6]<br>n=4	4.9[3]<br>[3.1-6.3]<br>n=15	2.6[3]<br>[2.4-2.8]<br>n=3
	**+CF**	**MPN^{+CF}/CFU**<br>median [IQR]	2.4<br>[2.4-6.9]<br>n=3	6.5<br>[4.8-314]<br>n=4	5.9<br>[3.5-8.0]<br>n=14	6.2<br>[5.4-11.9]<br>n=4
**DD Mtb negative samples**	**All**	**MPN/CFU**<br>median [IQR]	1.1<br>[0.8-1.4]<br>n=50	1.2<br>[0.94-1.4]<br>n=27	0.9<br>[0.71-1.7]<br>n=17	1.1<br>[0.78-1.5]<br>n=26
	**-CF**	**MPN^{-CF}/CFU**<br>median [IQR]	1.1<br>[0.94-1.4]<br>n=24	1.2<br>[0.92-1.4]<br>n=14	0.7<br>[0.51-1.0]<br>n=8	1.0<br>[0.35-1.6]<br>n=14
	**+CF**	**MPN^{+CF}/CFU**<br>median [IQR]	1.0<br>[0.80-1.5]<br>n=26	1.2<br>[1.1-1.6]<br>n=13	1.3<br>[0.92-1.7]<br>n=9	1.30<br>[1.2-1.4]<br>n=12

p-value for DS vs DR: [1] .094; [2] .010; [3] .086; IQR, InterQuartile Range

Table S7. Summary of Mtb counts in sputum from subjects with drug sensitive (DS) or drug resistant (DR) TB at day 0 and week 2 post-initiation of therapy.

		Day 0			Week 2		
		DS	DR	p-value[1]	DS	DR	p-value[2]
All subjects	**Log_{10} CFU** Median [IQR]	6.54 [6.05-6.85] n=29	6.23 [5.24-6.67] n=18	0.078	3.57 [3.09-4.47] n=23	3.39 [1.62-4.67] n=17	0.404
	**Log_{10} MPN^{-CF}** Median [IQR]	6.57 [6.21-6.89] n=29	6.34 [5.65-6.77] n=19	0.139	4.21 [3.13-5.13] n=23	3.65 [1.08-4.75] n=18	0.312
	**Log_{10} MPN^{+CF}** Median [IQR]	6.44 [6.05-6.76] n=29	6.53 [5.74-6.86] n=18	0.470	4.34 [3.32-5.09] n=23	4.31 [2.51-5.05] n=17	0.702
	**MPN^{-CF}/CFU** Median [IQR]	1.2 [0.95-1.6] n=29	1.3 [0.94-1.48] n=18	0.887	2.9 [1.2-5.5] n=23	1.1 [0.36-1.95] n=17	0.010
	**MPN^{+CF}/CFU** Median [IQR]	1.1 [0.8-1.5] n=29	1.6 [1.2-1.9] n=17	0.094	3.2 [1.7-6.7] n=23	1.4 [1.2-2.4] n=16	0.040
	**MPN^{+CF}/MPN^{-CF}** Median [IQR]	0.8 [0.55-1.2] n=29	1.3 [0.71-1.8] n=18	0.032	1.3 [0.91-1.6] n=23	1.4 [1.0-2.7] n=17	0.256
DD Mtb positive subjects	**Log_{10} CFU** Median [IQR]	6.06 [5.73-6.37] n=6	3.47 [3.36-5.11] n=5	0.018	3.57 [3.09-4.57] n=15	4.26 [1.77-4.69] n=5	0.896
	**Log_{10} MPN^{-CF}** Median [IQR]	6.51 [6.36-6.77] n=6	3.77 [3.71-5.54] n=5	0.011	4.31 [3.75-5.43] n=15	4.67 [1.19-5.05] n=5	0.662
	**Log_{10} MPN^{+CF}** Median [IQR]	6.32 [6.11-6.39] n=6	5.31 [4.34-5.71] n=5	0.067	4.44 [3.61-5.17] n=15	5.05 [3.23-5.48] n=5	0.930
	**MPN^{-CF}/CFU** Median [IQR]	2.6 [2.5-3.3] n=6	2.5 [2.3-2.7] n=5	0.715	4.9 [3.1-6.3] n=15	2.3 [2.1-2.4] n=5	0.010
	**MPN^{+CF}/CFU** Median [IQR]	2.0 [1.4-2.4] n=6	5.7 [2.3-7.4] n=5	0.273	5.0 [3.3-7.7] n=15	6.1 [4.7-6.2] n=5	0.827
	**MPN^{+CF}/MPN^{-CF}** Median [IQR]	0.68 [0.55-0.73] n=6	2.3 [1.0-5.8] n=5	0.068	1.0 [0.7-1.2] n=15	2.4 [1.7-2.7] n=5	0.126
DD Mtb negative subjects	**Log_{10} CFU** Median [IQR]	6.60 [6.07-6.93] n=23	6.57 [5.94-6.82] n=13	0.780	3.68 [2.90-4.41] n=8	3.18 [1.60-4.41] n=12	0.643
	**Log_{10} MPN^{-CF}** Median [IQR]	6.57 [6.08-6.89]	6.51 [6.05-6.83]	0.705	3.04 [2.46-4.25]	3.40 [0.99-4.67]	0.758

	Day 0			Week 2		
	**DS**	**DR**	**p-value[1]**	**DS**	**DR**	**p-value[2]**
	n=23	n=13		n=8	n=12	
**Log_{10} MPN^{+CF}** Median [IQR]	6.51 [6.13-6.88] n=23	6.74 [6.08-6.89] n=12	0.689	3.24 [2.82-4.36] n=8	3.54 [1.84-4.50] n=11	0.934
**MPN^{-CF}/CFU** Median [IQR]	1.1 [0.94-1.4] n=23	1.1 [0.92-1.4] n=13	0.531	0.73 [0.51-1.0] n=8	1.0 [0.34-1.4] n=12	0.396
**MPN^{+CF}/CFU** Median [IQR]	1.0 [0.79-1.4] n=23	1.2 [1.1-1.6] n=12	0.251	1.3 [0.89-1.7] n=8	1.2 [1.0-1.4] n=11	0.804
**MPN^{+CF}/MPN^{-CF}** Median [IQR]	0.88 [0.61-1.2] n=23	1.2 [0.83-1.4] n=12	0.289	1.3 [1.0-1.6] n=8	1.2 [1.0-1.8] n=12	0.508

[1] Comparing DS vs DR at day 0; [2] Comparing DS vs DR at week 2; IQR, InterQuartile Range

Table S8. Rate of change in numbers of viable Mtb from day 0 to week 2 in sputum from subjects with drug sensitive (DS) and drug resistant (DR) TB.

	**DS (n=23)**	**DR (n=15-18)**	**p-value[1]**
**∆ Log10 CFU** Median [IQR]	-2.27 [-3.20, -1.97]	-2.63 [-3.29, -2.15]	0.436
**∆ Log10 MPN^{-CF}** Median [IQR]	-2.00 [-2.97, -1.18]	-2.82 [-3.71, -2.13]	0.106
**∆ Log10 MPN^{+CF}** Median [IQR]	-1.92 [-2.82, -1.22]	-2.41 [-3.37, -1.79]	0.199
**∆ MPN^{-CF}/CFU** Median [IQR]	2.1 [0.74, 4.3]	0.84 [0.59, 1.8]	0.034
**∆ MPN^{+CF}/CFU** Median [IQR]	2.7 [1.2, 5.6]	0.83 [0.60, 1.5]	0.013
**∆ MPN^{+CF}/ MPN^{-CF}** Median [IQR]	1.2 [0.87, 2.2]	1.1 [0.74, 3.3]	0.627

[1] Comparing DS to DR; IQR, InterQuartile Range
